# Analgesic Efficacy of Diclofenac and Paracetamol vs. Meperidine in Cesarean Section

**DOI:** 10.5812/aapm.9997

**Published:** 2013-12-26

**Authors:** Heidar Darvish, Behrouz Memar Ardestani, Sara Mohammadkhani Shali, Ali Tajik

**Affiliations:** 1 Anesthesiology Department, Amiralmomenin Hospital, Islamic Azad University, Tehran Medical Branch, Tehran, IR Iran; 2 Department of Medical Researches, Islamic Azad University, Tehran Medical Branch, Tehran, Iran; 3 Department of Community Medicine, Tehran University of Medical Sciences, Tehran, Iran

**Keywords:** Diclofenac, Acetaminophen, Meperidine, Cesarean Section, Pain, Postoperative

## Abstract

**Background:**

One of the most important complications in cesarean surgery is postoperative pain, and different ways have been proposed to control it.

**Objectives:**

The purpose of this study was to determine the efficacy of Diclofenac and Paracetamol combination in comparison with Meperidine on postoperative pain after cesarean surgery.

**Patients and Methods:**

One hundred and twenty women candidates for elective cesarean section under spinal anesthesia categorized as ASA class I were selected and randomly assigned to receive either Diclofenac suppository at the end of the operation and thereafter 1 gram infused bolus of Paracetamol (group A), or 20 mg bolus of Meperidine after transition to recovery room (group B) to control postoperative pain.

**Results:**

Postoperative pain was present in recovery in 38.3% and 23.3% in groups B and A, respectively (P = 0.009). Postoperative pain was seen after six hours of operation in 38.7% and 16.7% in groups B and A, respectively (P = 0.010). Postoperative pain was present after 12 hours of operation in 38.3% and 15% in groups B and A, respectively (P = 0.002). The additive Meperidine use was the same between the two groups in recovery (P > 0.05). The additive Meperidine use was seen after six hours of operation in 26.7% and 6.7% in groups B and A, respectively (P = 0.013). The additive Meperidine use was seen after 12 hours of operation in 16.7% and none of the patients in groups B and A, respectively (P = 0.004). The frequency of drug adverse effects was the same between the two groups (P > 0.05).

**Conclusions:**

Totally, according to the obtained results it may be concluded that Paracetamol and Diclofenac combination would have a better efficacy in postoperative pain control and need reduction to additive analgesia compared to Meperidine.

## 1. Background

Cesarean section is one of the main surgeries performed in obstetric/gynecologic departments which its rate is increasing due to various causes including rise in marital age, legal issues in the obstetric/gynecologic department, socioeconomic status of the community, and etc. (1). Therefore, cesarean section is one of the health priorities of the community (1), and management of postoperative complications is of great importance (2).

One of the main complications of cesarean surgery is postoperative pain which patients are faced with (1). Postoperative pain management is an important component of adequate postoperative patients care in all surgical procedures (3) including obstetric operations (4). Other than causing an unpleasant feeling, pain increases the time needed to get out of bed, duration of hospitalization, immobility and patient’s reduced desire to move, and also complications caused by immobility such as atelectasia, deep vein thrombosis, and constipation (2). Hence, pain management is an important part of every hospitalization course especially those accompanied with invasive procedures (5). On the other hand pain intensity is in direct association to inflammatory system activity and cytokines levels which create complications in patients; however, pain relief can result in very good results (2). Therefore pain management, as one of the main factors of postoperative care, has always been noteworthy to anesthesiologists (6, 7).

Several methods including pharmacological and anesthesiological approaches are available for pain relief after cesarean section (8-10). Each has specific performance and effectiveness, and therefore various studies need to be conducted to determine and compare the effectiveness of these different methods. 

## 2. Objectives

Therefore, in this study, the efficacy of nonopioid drugs such as nonsteroidal anti-inflammatory drug (Diclofenac) and Acetaminophen were compared with narcotics (Meperidine). So if its effectiveness would be approved in controlling postoperative pain, narcotic medication side effects such as dyspnea, nausea and vomiting, itching and urinary retention could be avoided in postoperative pain management.

## 3. Patients and Methods 

One hundred and twenty women with anesthesia class of ASA-I were selected among those undergoing elective cesarean section under spinal anesthesia at Amiralmomenin hospital, Tehran, Iran between 2011 and 2012, and randomly divided into two groups (N = 60). Exclusion criteria were general anesthesia during cesarean section for any reason, complications during cesarean section, and allergic reactions to medications. This study was approved by the Ethical Committee of Islamic Azad University, Tehran Medical Branch, and the Helsinki Declaration was respected all over the study course.

All cases received 10 ml of ringer-lactate serum through appropriate IV line. After monitoring blood pressure, heart rate, heart rhythm, oxygen saturation, getting 5-6 liter per minute of oxygen by mask, and sitting in preparation for spinal anesthesia in L4-L5 intervertebral space, 2-2.5 milliliter of Marcaine 0.5% (Bupivacaine) was injected to L3-L4 space through a 26 gauge needle. In case of blood pressure reduction, ringer-lactate and ephedrine were administered. For postoperative pain control in group A, two rectal suppositories of Diclofenac were used after completion of cesarean section and on the operating table. One gram bolus of Paracetamol (1 gram of Paracetamol diluted in 100 milliliter of normal saline) was infused within 15 minutes while transferring to the recovery room. The patients then received the above mentioned dose repeatedly every four hours for the next 12 hours. On the other hand, if group A patients had moderate pain they received 10 mg of Meperidine intravenously.

A bolus of 20 mg of Meperidine was infused immediately after transferring to recovery room in group B patients and received a repeated dose of 20 mg every four hours for the next 12 hours. More than four (moderate and severe pain) patients received an extra dose of 10-20 mg of Meperidine due to severe pain between Meperidine injections based on visual analogue scale (VAS). The variables were age, duration of surgery, side effects and pain according to VAS. The results were analyzed using SPSS version 13.0, and pain intensity and Meperidine consumption were compared between the two groups. For qualitative variables, frequency and percentage frequency and for quantitative variables mean and standard deviation were reported. The T-test was used for evaluation of differences between the two groups with a statistically significant difference at 0.05.

## 4. Results

The mean age and duration of operation were alike between the two groups (Table 1). Postoperative pain was present in recovery in 38.3% and 23.3% in Meperidine group and Diclofenac/Paracetamol group, respectively (P = 0.009). Postoperative pain was present after six hours of operation in 38.7% and 16.7% in Meperidine group and Diclofenac/Paracetamol group, respectively (P = 0.010). Postoperative pain was present after 12 hours of operation in 38.3% and 15% in Meperidine group and Diclofenac/Paracetamol group, respectively (P = 0.002). 

**Table 1.** Mean age and Duration of Operation in the Two Groups

Variable	Mean ± SD
**Age, y**	
Paracetamol and Diclofenac	27.67±4.24
Meperidine	27.25±4.87
**Duration of operation**	
Paracetamol and Diclofenac	1.70±0.76
Meperidine	1.58±0.72

Additive Meperidine use was the same between the two groups in recovery (P > 0.05). Additive Meperidine use was seen after six hours of operation in 26.7% and 6.7% in Meperidine group and Diclofenac/Paracetamol group, respectively (P = 0.013). Additive Meperidine use was seen after 12 hours of operation in 16.7% and none of the patients in Meperidine group and Diclofenac/Paracetamol group, respectively (P = 0.004). The frequency of drug adverse effects was the same between the two groups (Table 2). 

**Table 2.** Frequency of Drug Adverse Effects in the Two Groups

Group	Drug adverse effects		
	None, No. (%)	Itching, No. (%)	Nausea and Vomiting, No. (%)
**Paracetamol and Diclofenac**	52 (86.7)	1 (1.7)	7 (11.7)
**Meperidine **	49 (81.7)	1 (1.7)	10 (16.7)

## 5. Discussion

Cesarean section constitutes a public health priority (1, 11) because it is one of the main surgeries in the obstetrics and gynecologist wards and its rate is increasing due to increased marital age, legal issues in obstetric/gynecologic ward, and socio-economic status of the community, and prevention of its postoperative complications is of great importance (2). One of the main postoperative complications of cesarean is pain (3). Several methods are available for pain relief after cesarean surgery (12, 13), each with its performance and efficacy. Therefore in this study we compared the pain reduction effects of Diclofenac suppository and Paracetamol compared to intravenous Meperidine after the operation under spinal anesthesia. Results of this study indicated that combination therapy especially with using an analgesic with central effect similar to Paracetamol would have a greater efficacy than single-therapy without increasing the rate of complications.

In a study conducted by Gleeson and colleagues in England it was declared that Meperidine use caused analgesia in 87% of patients at postoperative phase (14), but in our study this rate was 61.7% at the end of the 12 hours which is less than the results obtained in the mentioned study which could be due to the shorter follow-up period in our study in comparison with them. In a study conducted by Siddik and colleagues in London it was declared that Meperidine has good effects on pain relief after cesarean section (15). In our study also Meperidine had a good effect in postoperative pain reduction in three of every five patients (about 60%). In a study conducted by Davis et al. in the United States, it was declared that using Paracetamol caused significant postoperative pain reduction (16), which in this study this rate was 87% in Paracetamol and Diclofenac combination therapy.

In a study conducted by Kilicaslan and colleagues in Turkey it was declared that Paracetamol increases analgesia and reduces the need to Tramadol (17) which is consistent with our findings in the current study. In a study conducted by Munishankar et al. in England it was declared that simultaneous use of Paracetamol and Diclofenac caused 38% reduction in the Morphine use in comparison to the use of Paracetamol alone (18), which in our study combination therapy was significantly more effective than single-therapy. In a study conducted by Remy and colleagues it was declared that Acetaminophen use induced analgesic effect of Morphine postoperatively without changing the incidence of postoperative complications (19). This synergic drug reaction in postoperative pain reduction by analgesic use was observed in our study using Diclofenac and Paracetamol.

In Wilder-Smith and associates study the effect of Diclofenac and Tramadol use in pain reduction after cesarean surgery was evaluated in 120 cases, and it was found that the effect of the two intramuscular drugs including Diclofenac 75 mg and Tramadol 100 mg simultaneously was significantly over the effects of each one alone in reducing pain after cesarean (20), which the greater efficacy of combination therapy was approved in our study compared with single-therapy. In a research by Ong et al. it was concluded that combined use of Paracetamol and a nonsteroidal anti-inflammatory drug may have a greater analgesic effect compared with using each one separately (21), which is consistent with our findings.

In a study in Sweden conducted by Legeby and colleagues on 50 women undergoing mastectomy, it was found that using Diclofenac suppositories with a dose of 100 mg reduced narcotic drugs consumption significantly up to 34% compared to placebo (22), which our study also revealed the good effectiveness of Diclofenac use in combination with Paracetamol. In a study conducted by Fayaz et al. on 60 patients in England, it was found that using Diclofenac suppository with a dose of 100 mg as well as a combination of Diclofenac and Paracetamol had a significant effect on reducing the need for narcotic drugs use in comparison with placebo (23), which is in line with our study findings.

In a study by Sylaidis and colleagues conducted on twenty patients in England it was stated that a single 100 mg rectal dose of Diclofenac had a good effect on reduction of postoperative pain and the need to use other narcotic analgesics (24) which complies with our study findings. Also another study by Hosseini Jahromi et al. (25) evaluated the effects of suppository Acetaminophen, Bupivacaine Wound Infiltration, and Caudal Block with Bupivacaine on postoperative pain in management of inguinal herniorrhaphy in children, and it was seen that bupivacaine infiltration and caudal block with bupivacaine provide better analgesia than suppository acetaminophen. However in our study also the combination treatment resulted in a better analgesic response.

Totally, according to the results of this study and comparison with other studies performed in this field of health, it may be concluded that combination of Diclofenac and Paracetamol effectiveness is far better at reducing pain after cesarean section and the amount of required analgesics compared to Meperidine. However further studies are required to confirm and validate the findings obtained in the current study.
